# Are Children with Autism Spectrum Disorder Initially Attuned to Object Function Rather Than Shape for Word Learning?

**DOI:** 10.1007/s10803-015-2657-5

**Published:** 2015-12-14

**Authors:** Charlotte Field, Melissa L. Allen, Charlie Lewis

**Affiliations:** Department of Psychology, Fylde College, Lancaster University, Lancaster, LA1 4YF UK

**Keywords:** Autism spectrum disorder, Developmental disorder, Function bias, Shape bias, Word learning

## Abstract

We investigate the function bias—generalising words to objects with the same function—in typically developing (TD) children, children with autism spectrum disorder (ASD) and children with other developmental disorders. Across four trials, a novel object was named and its function was described and demonstrated. Children then selected the other referent from a shape match (same shape, different function) and function match (same function, different shape) object. TD children and children with ASD were ‘function biased’, although further investigation established that having a higher VMA facilitated function bias understanding in TD children, but having a *lower* VMA facilitated function bias understanding in children with ASD. This suggests that children with ASD are initially attuned to object function, not shape.

## Introduction

Typically developing (TD) children use both object shape (the ‘shape bias’, Landau et al. 1988) and function (the ‘function bias’, Gentner 1978) as a basis for lexical extension to other category members. The shape bias involves forming word-object mappings according to similarity in shape, such as calling an unfamiliar object a ‘ball’, due to its prototypical round form, rather than generalising object labels according to similarity in other perceptual characteristics, such as colour, texture or size. Although generally a useful heuristic, there are occasions where the shape bias could actually hinder word learning. An orange and a basketball are both spherical but different types of objects, while a beanbag chair may be round and an armchair may be larger and squarer shaped, despite being the same type of object. What unifies objects is not simply perceptual similarity, but the shared role they fulfil (Bloom 2004; Keleman 1999). Thus, a bias that constrains word-object mappings according to similarity in function can be adaptive (‘function bias’). An unfamiliar object is called a ‘ball’ not just because of its appearance, but also because of its role: to bounce, kick or throw.

TD children have been found to show a function bias when object shape and function conflict. When a novel object is named and its function is clearly described and demonstrated, children extend the label to a differently shaped object that shares the same function, rather than to a similarly shaped one with a separate function (e.g. Diesendruck et al. 2003; Merriman et al. 1993). This attention to function strengthens with chronological age (CA) in typical development, and may also be dependent on an individual’s non-verbal skills or language ability. Specifically, children have to notice that different objects share the same function, and that these objects also tend to share the same name, which may respectively recruit both these abilities. The role of language and non-verbal skills can be directly addressed by comparing performance of TD children with children who have different developmental trajectories in terms of these skills, particularly children with autism spectrum disorder (ASD).

Although four studies have directly addressed the absence of (see Hartley and Allen 2014; Potrzeba et al. 2015; Tek et al. 2008), or delay in acquiring (see Field et al. 2015), a shape bias in ASD, to our knowledge no research to date has investigated the function bias in this population. Thus, the current study aims to fill this gap in the literature. There are reasons to believe that children with ASD might show differences relative to TD children with respect to understanding object function. For instance, abundant evidence suggests that children with ASD often demonstrate idiosyncratic, stereotyped and restricted artefact use (Ozonoff et al. 2008; Wulff 1985). This includes repetitively spinning the wheels on a car or trickling sand and water between their fingers, lining objects up in rows or piling objects on top of each other, and spinning, rotating, rolling, mouthing and banging artefacts (Leekam et al. 2011; Ozonoff et al. 2008; Williams et al. 1999). These unusual responses to objects may hinder children’s discovery of the artefact’s proper function (Loveland 1991; Williams et al. 1999).

As children with ASD have weak central coherence and a preference for component parts rather than the object gestalt (Frith 1989; Happe and Frith 2006), they might be so fixated on manipulating the parts of objects that they fail to comprehend the overall role that objects fulfil. For example, repeatedly spinning the wheels on a toy car distracts the child from the car’s true function of driving. The function bias also involves attending to and remembering the function of new artefacts and comparing this information to previously stored knowledge about object functions. This may be difficult for children with ASD because of impairments with prototype formation (Klinger and Dawson 2001) and categorisation (Gastgeb et al. 2006, 2011). Furthermore, children with ASD experience referential intent difficulties (Prizant and Wetherby 1987) and the function bias has been linked to intentional understanding (Diesendruck et al. 2003).

Therefore, it is possible that children with ASD have a function bias delay (develop the function bias later than TD individuals) or deviance (fail to develop the function bias at all). Children with ASD exhibit delay or deviance in other areas of language acquisition (e.g. Bartolucci et al. 1976; Eigsti and Bennetto 2009; Howlin 1984; Mitchell et al. 2006; VanMeter et al. 1997) and are delayed showing a shape bias (Field et al. 2015). In order to establish if ASD involves a function bias delay or deviance, testing a cohort of children with wide variation in language ability is necessary.

Despite some studies suggesting a function bias deficit in ASD, other evidence suggests children with ASD might show the heuristic. For instance, they show other word learning constraints and biases, such as mutual exclusivity (Preissler and Carey 2005) and the noun bias (Swensen et al. 2007). They also classify objects by function to the same extent as their TD peers (Tager-Flusberg 1985; Ungerer and Sigman 1987). In Tager-Flusberg (1985), children viewed a test picture (e.g. a car) then a picture from the same category (e.g. a bus) and a distractor picture from a different category (e.g. an item of clothing). The children with ASD were able to correctly categorise not only perceptually similar objects (such as different types of dogs) but also functionally but not perceptually related objects (such as different types of furniture) into their correct category. Ungerer and Sigman (1987) also found that children with ASD categorised objects according to functional similarity (e.g. different animals, fruits, vehicles and furniture) as well as the more perceptually salient characteristics of colour and form. This suggests that children with ASD have some understanding that the same type of objects have the same function.

Children with ASD also partake in functional play (Baron-Cohen 1987; Leslie 1987; Libby et al. 1998; Ungerer and Sigman 1981), such as brushing one’s hair with a toy brush, holding a telephone to one’s ear and sweeping the floor with a toy broom. Functional play helps children name things, learn how to use objects appropriately and make associations between the roles of different artefacts (Mastrangelo 2009). Being able to classify objects by function and take part in functional play suggests that children with ASD have a basic level of understanding about the role objects fulfil.

Therefore, there is conflicting evidence regarding functional understanding in children with ASD. To examine whether any differences which may emerge in terms of showing the function bias in ASD relative to TD children are simply a result of cognitive delay, rather than ASD per se, it is necessary to also examine the function bias in children with other developmental disorders (DD). Like children with ASD, children with DD categorise objects by the function they fulfil (Ungerer and Sigman 1987) and engage in functional play (Malone and Langone 1998; Sigafoos et al. 1999), suggesting they have some functional understanding.

However, there is mixed evidence for the use of word learning constraints in this population. Some children with DD show a shape bias in naming contexts (Field et al. 2015) and use mutual exclusivity for novel word learning (Wilkinson and Albert 2001; Wilkinson 2005). Other studies report that children who are ‘late talkers’ have a shape bias deficit (Jones 2003), and children with intellectual disability have difficulty with fast mapping and are less able than TD children to maintain labels when tested 1–3 days later (Wilkinson 2005). Thus, testing children with DD can inform theories of language acquisition in this population, as well as elucidate whether potential differences in ASD stem from cognitive delay.

To investigate the function bias, we based our task on Diesendruck et al. (2003), who found that 3-year-old TD children form word-object mappings by function rather than shape, but only when object function is explicitly described and demonstrated. In the ‘label + intended function’ condition, participants were presented with a novel object, which was labelled and its function was clearly articulated and demonstrated to the children. For example, the experimenter stated *‘this is a wug and it can hold coins’* and then poured some coins into the object. The function of the novel object and the two test objects were also described and demonstrated (i.e. it was made explicitly clear to the children that the shape match was the same shape as the novel object but performed a different function, while the function match was a different shape but performed the same function). When asked to give the experimenter the other *‘wug’*, the children chose the function match test object.

Although we kept the procedure of our study the same as Diesendruck et al. (2003), we recruited a large sample of participants of varying ages, due to the controversy within the TD literature regarding the precise age of function bias onset. It is generally agreed that by adulthood TD individuals show a function bias rather than shape bias when shape and function conflict (Graham et al. 1999; Jones 1998; Landau et al. 1998), however it is unknown at what age this ability appears. Although Diesendruck et al. (2003) claim that TD children show a function bias at 3-years-old (see also Kemler-Nelson et al. 2000, who found a function bias in 4-year-old children and Kemler-Nelson et al. 2000 who found a function bias in 2-year-olds), others argue that the function bias does not develop until age 6 (Merriman et al. 1993) or even later (Gathercole and Whitfield 2001).

It is predicted that TD and DD children will override the shape bias in favour of a function bias, replicating Diesendruck et al. (2003). Our first hypothesis is that TD and DD children will show a clear understanding of the latter. We expected that children with ASD would show a function bias deficit, due to idiosyncratic object use. However, as children with ASD categorise objects by function and engage in functional play, an alternative possibility is that they show a function bias. It is also possible that children’s performance on Diesendruck’s task is related to the child’s language level, replicating Merriman et al. (1993). Our second hypothesis is therefore that the function bias does not develop until children have reached a higher level of receptive understanding.

## Method

### Participants

One-hundred-and-twenty-four children were recruited (see Table 1 for the background and test data from the three groups of participants). The participants were recruited from four mainstream and 12 specialist schools, one ASD class within a mainstream school, two parental support groups and 3 day nurseries across the North West of England and from a database of parents who had previously expressed an interest in their children participating in psychology research at Lancaster University. Participants were matched according to the group mean verbal mental age (VMA). Although the DD children had a slightly lower VMA than the other two groups (see Table 1), a one-way ANOVA showed that this was not significant. The DD children had various conditions, primarily intellectual disability and rare chromosomal disorders. Lancaster University Research Ethics Committee granted ethical permission for the study to take place. Written informed consent was obtained from children’s parent or guardian.

**Table 1:** Background information and mean proportion of function match responses for the three groups of participants

	TD N = 45, 22 males	ASD N = 51, 45 males	DD N = 28,15 males
Mean CA (SD)	4.63 (1.44)	9.60 (3.35)	9.27 (2.32)
Range	2.00–7.00	4.33–17.42	5.17–15.58
Mean VMA (SD)	5.33 (2.08)	5.25 (1.98)	4.43 (1.84)
Range	2.75–11.67	2.25–11.58	2.33–8.83
Mean Ravens score (SD)	13.93 (7.44)	18.39 (8.49)	10.58 (6.88)
Range	4.00–32.00	0.00–36.00	2.00–31.00
Mean CARS score (SD)	16.36 (2.16)	34.70 (7.48)	24.18 (4.93)
Range	15.00–22.50	20.00–53.00	16.00–32.00
Mean SCQ score (SD)	2.47 (3.73)	18.65 (6.70)	7.00 (5.26)
Range	0.00–12.00	5.00–34.00	1.00–21.00
Function bias score (SD)	.66 (.37)	.56 (.33)	.45 (.29)
Range	0–1	0–1	0–1

### Cognitive Tests

The British Picture Vocabulary Scale—Second Edition (BPVS-II; Dunn et al. 1997) was administered to determine children’s VMA.^1^ Raven’s Coloured Progressive Matrices (Raven’s 2003) was administered to determine children’s nonverbal reasoning abilities. The Raven’s has a minimum raw score of 0 and a maximum of 36.

### Clinical Diagnoses

All children with ASD had received a prior clinical diagnosis of autism by a qualified educational or clinical psychologist, using standardised instruments (i.e. Autism Diagnostic Observation Scale and Autism Diagnostic Interview—Revised: Lord et al. 1994, 2002) and expert clinical judgment. Inclusion in the final sample was based on these specialist diagnoses, derived from the DSM-IV-TR. However, for most children, the Childhood Autism Rating Scale (CARS; Schopler et al. 1988) and the lifetime version of the Social Communication Questionnaire (SCQ; Rutter et al. 2003) were also completed by a parent or teacher (CARS: 21 TD, 46 ASD, 19 DD. SCQ: 19 TD, 46 ASD, 22 DD) in order to provide an additional characterisation of the sample. Scores on the CARS range from 15 to 60, with scores of 30 + in the ASD range. Scores on the SCQ range from 0 to 39, with scores of 15 + in the ASD range. The vast majority of children scored according to their diagnosis on the scales with just four children (3 ASD, 1 DD) not scoring according to their diagnosis on either questionnaire. As excluding these children from the analyses yielded almost identical results, these participants were included in the final sample.

With two exceptions, all of the DD children had received a formal diagnosis of their disorder. The data from the remaining two DD children were not excluded from the study because, in addition to attending a specialist school, their VMA (3.67 and 3.75 respectively) was considerably younger than their CA (10.75 and 10.83 respectively). The possibility that these children had undiagnosed ASD was ruled out by both children scoring below the clinical threshold for ASD on both the CARS and SCQ questionnaires.

### Materials

**Fig. 1:**
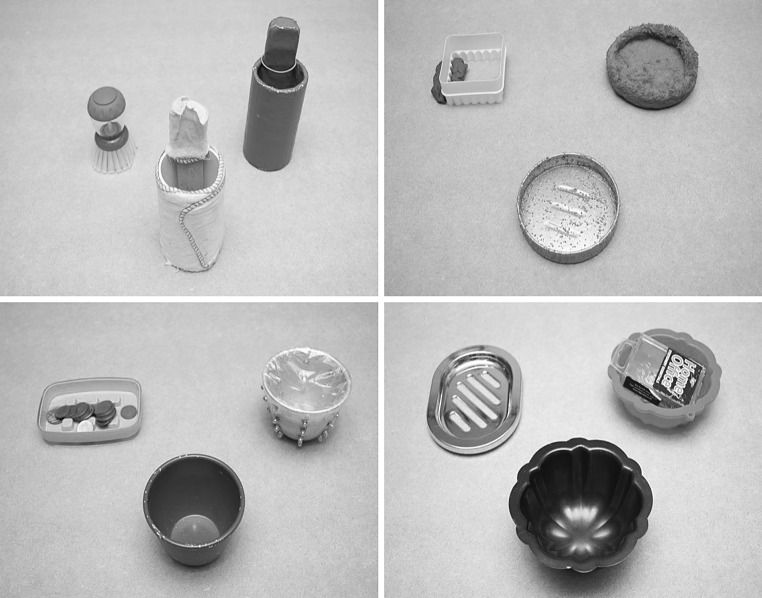
The four object sets. Novel objects (*centre*) (from *left* to *right*, with designated function in brackets): puppet stand covered with Mr. Sheen duster (dusting), *silver sandpaper* covered soap dish (cutting playdough), unaltered* green bowl* (holding coins), *black jelly* mould (making music). Function match test objects (*left*): dish brush, cutter, soap holder, soap dish. Shape match test objects (*right*) (with designated function in brackets): puppet stand painted *blue* (hanging hair ties), soap dish covered with* blue* towel (mopping up water), bowl with plastic cover around the top (sticking), *red jelly* mould (holding paperclips) (Color figure online)

A total of twelve objects were presented to the children across four trials (see Fig. 1). The functions of the objects largely followed those used by Diesendruck et al. (2003). However, there were some minor adaptations, in order to make the study more culturally relevant. For example, the function of ‘cutting clay’ was changed to ‘cutting playdough’.

Diesendruck et al. (2003) included within their study several objects where the name would already be familiar to the children (e.g. a solid wooden block, a rectangular box and a piece of wood), alongside more novel items (e.g. hanger-like shapes made out of pipe cleaner and wire, a round disk made out of felt). In line with this, some of our objects were more familiar to the children than others, although the objects were used to perform functions that they were not typically associated with. No child in our study volunteered a name for any of the stimuli.

### Procedure

Participants completed the task individually in a quiet place within their school, day nursery, parental support group or the Centre for Research in Human Development and Learning (CRHDL), Lancaster University. The methodology followed Diesendruck et al. (2003), replicating their dialogue when introducing the novel object, function match and shape match. The experimenter presented the novel object and stated *‘this is a jop (cheem/kiv/glire) and it was made for cutting playdough (holding coins/dusting/making music). See how it cuts playdough (holds coins/dusts/makes music)’*. The experimenter then demonstrated this function, by producing some playdough and cutting it with the object (pouring a selection of coins into the object/moving the object around on the table in a dusting motion/banging a highlighter against the object to make a sound) and then placed it upon the table.

Introducing the function match test object, the experimenter said *‘see this one? It can cut playdough because it was made for cutting playdough.’* The experimenter demonstrated this function, by cutting the playdough, then continued *‘see, it doesn’t look like this one* [pointing to the original], *they have a different shape. It can cut playdough because it was made for cutting playdough.’* The experimenter demonstrated this function for a second time. Introducing the shape match test object, the experimenter said *‘see this one? It can’t cut playdough because it was made for mopping up water (sticking/hanging hair ties/holding paperclips)’.* The experimenter demonstrated this function, by pouring a tiny amount of water onto the table and mopping it up, then continued *‘See, it looks like this one* [pointing to the original], *they are the same shape. It can’t cut playdough because it was made for mopping up water’* [demonstrating this function for the second time].

Following this, the experimenter picked up the novel object and said *‘remember I told you that thisis a jop and it was made for cutting playdough. One of these* [pointing to the test objects] *is also a jop. Which one of these is a jop?’* The word uttered to refer to the novel object, the order that the test objects were presented, the order that the function match and shape match were introduced and the positioning of the test objects on the table (left or right) were all counterbalanced. One TD child, two children with ASD and one DD child only completed three out of the four trials and one TD child only completed one out of the four trials, due to inattention.

## Results

The data were analysed in three ways. Firstly, following Diesendruck et al.’s (2003) non-parametric approach, we classed participants as ‘function biased’ (selected the function match for three or four trials), ‘shape biased’ (selected the shape match for three or four trials) or ‘not biased’ (selected the function match and shape match for two trials each) (see Table 2). One sample Chi Square analyses showed that the TD children (23/44) and those with ASD (24/51), but not children with DD (7/28), were function biased at a rate above chance [TD, χ^*2*^(2, *N* = 44) = 11.77, *p* = .003, *w* = .49; ASD, χ^*2*^(2, *N* = 51) = 8.50, *p* = .014, *w* = .41; DD χ^*2*^(2, *N* = 28) = 3.01, *p* = .22].

**Table 2:** Percentage of children who were function biased, shape biased and not biased using Diesendruck’s scoring procedure

	TD	ASD	DD
Function biased	53.30**	47.10*	25.00
Shape biased	28.90	33.30	46.40
Not biased	17.80	19.60	28.60

* *p* < .05; ** *p* < .01

**Fig. 2:**
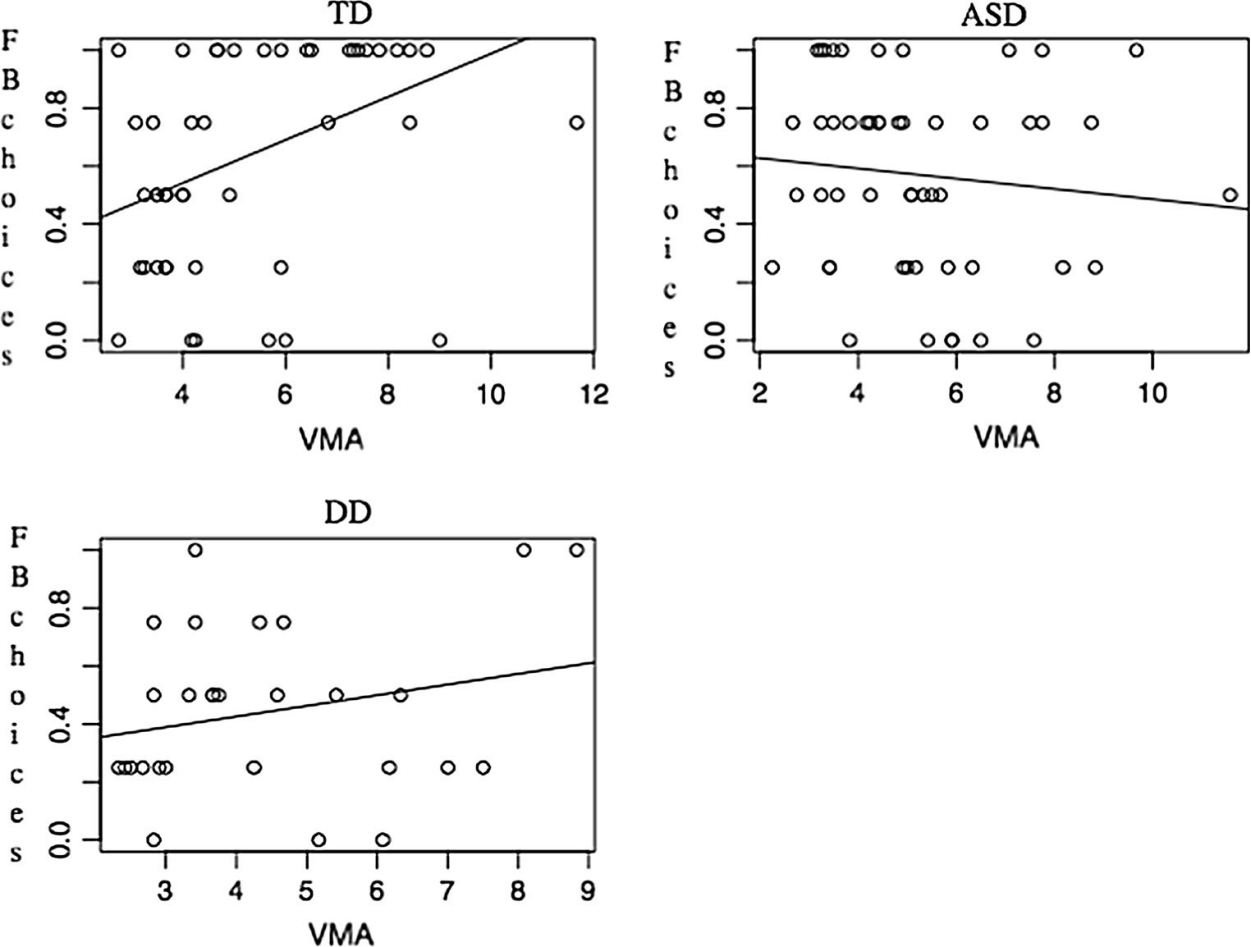
Scatterplots of VMA by function match responses

We then conducted a series of logistic regressions on whether the children were function biased or not using VMA, Raven’s, and group membership as explanatory factors. The saturated model was significant and we therefore extracted variables to find the best-fit model. This showed that the model was highly significant [χ^*2*^(4) = 19.22, *p* = .001; *Nagelkerke R*^*2*^ = .19]. There were main effects for Group [Wald(*2*) = 8.43, *p* = .015] with follow up analysis showing that this was explained by a significant difference between the TD and DD groups [Wald(*1*) = 4.2, *p* = .04]. In addition there was a VMA by Group interaction [Wald(*2*) = 9.77, *p* = .008], accounted for by a difference between ASD and TD children in terms of their language levels [Wald(*1*) = 8.24, *p* = .004] (see Fig. 2). This analysis suggests that the likelihood of being classified as function biased is determined by group membership and language level in different ways. For TD children, higher VMA facilitates function bias understanding. In contrast, for children with ASD, *lower* VMA facilitates function bias understanding.

Our second set of analyses used a parametric approach in order to explore more fully the relationship between performance on the series of test trials with regards to group membership (TD, DD and ASD) and the background factors (VMA and Raven’s) as continuous covariates. We averaged the four trials to construct a proportional scale of success on the function bias questions, with a range of 0–1. Proportions were used instead of frequencies as five children did not complete all trials. Preliminary investigations of the data suggested that the children’s VMA (as measured by the BPVS) appeared to relate to their performance on the function bias task, with the direction of this relationship varying according to group. In the TD and DD samples the two sets of scores appear to be positively related, while in the ASD children there seemed to be a slight negative relationship (see Fig. 2).

We conducted a linear mixed effects model using Group (TD, ASD, DD) as a fixed factor and BPVS scores and Raven’s test data as continuous covariates. This showed no main effects (Group: *F*(2,32) = .84; Raven’s: *F*(1,25) = 2.45; VMA: *F*(59,24) = .52, all *NS*). There was a Group × VMA interaction (*F*(24,35) = 2.03, *p* = .042, *η*_*p*_^*2*^ = .66). We followed this up by performing for each group a linear regression to examine the relationship between VMA and function bias scores. These showed that for the children with ASD and DD children the slopes were non-significant (*F* < 1.6), but for the TD children there was a clear association between the two scores (*F*(.43) = 6.88, *p* = .012: *B* = .07 (*SE* = .03), *Beta* = .37) (see Fig. 2). This suggested that the interaction is accounted for by the association in the TD children and not the two other groups.

Finally, we conducted three further checks on the data. First, unsurprisingly, there were far more males in the ASD group than the other two groups, which is reflective of the fact that more males than females are diagnosed with ASD (e.g. Fombonne 2003). However, as it is unknown whether gender has an effect on children’s function bias responses, following Hartley and Allen (2014), we carried out two follow up analyses to test for any gender effects. A 3 (Group: TD, ASD, DD) × 2 (Gender: Male or Female) Factorial ANOVA revealed no effect of Gender. We also re-ran all analyses including only the male children for the TD participants and replicated our findings.

Secondly, we wanted to establish if children’s object selection differed across object sets, as the novel objects for the ‘holding coins’ (green bowl) and ‘making music’ (jelly mould) trials might have been more familiar to the children than the novel objects for the ‘dusting’ (duster) and ‘cutting playdough’ (playdough cutter) trials. Despite this replicating Diesendruck et al. (2003), which contained a mixture of familiar and unfamiliar stimuli, we wanted to ensure that children were responding the same for the ‘familiar’ and ‘novel’ object sets. A paired samples *t* test found no significant differences between children’s responses for the two ‘novel’ compared with the two ‘familiar’ object sets for any of the three groups.

As a third precaution, we investigated if children’s responses were consistent across all object sets. Children’s responses were categorised for each trial separately as ‘shape match’ or ‘function match’. A Friedman test confirmed that there were no significant differences in terms of children’s responses per object set for any of the three groups.

## Discussion

Function plays an important role in children’s artefact categorisation. However, the exact age at which function overrides shape in children’s naming generalisations, is debateable, with some studies suggesting 3-years-old (e.g. Diesendruck et al. 2003), although others argue six (e.g. Merriman et al. 1993). Furthermore, the function bias has never before been explored in atypically developing participants, such as children with ASD. The tests against chance performance suggest that TD children and participants with ASD show a function bias. In keeping with the idea that this effect emerges in development, TD children appear to appreciate the role of functional information for lexical extension with more proficient receptive language ability. This is not the case for children with ASD, who may have a different route to word learning; forming word-object mappings by function to begin with. The emergence of the function bias in TD children will be considered first. The results for the DD children reveal a fundamental difficulty with function understanding and inform us about the role of cognitive delay in ASD. We will explore the DD findings before looking specifically how function might facilitate language acquisition in children with ASD.

The results do not directly contradict Diesendruck et al. (2003), who found that 3-year-old TD children showed a function bias. Nevertheless, the TD participants in this study, on average, possessed a higher CA (over four) and VMA (over five) than the 3-year-olds recruited in the former study. Our findings suggest that attention to the functional qualities of objects gradually develops, and are consistent with findings that TD children do not generalise according to function until they are older than three (e.g. Graham et al. 1999; Imai et al. 1994; Tomikowa and Dodd 1980; Matan and Carey 2001; Merriman et al. 1993). The likely conclusion here is that the function bias is truly slow to emerge across typical development given the focus on shape and other features of a perceptual array in early language acquisition (e.g. Horst and Twomey 2013; Landau et al. 1992, 1988; Tek et al. 2012).

A surprising feature of the results is that DD children did not use function for word-object mapping across the range of VMAs that we explored. It is possible that the language used within the procedure was too complex for DD participants. Following Diesendruck et al. (2003), the paradigm contained detailed verbal instruction, and children had to retain the pairings between objects and corresponding function in working memory. We chose to remain faithful to the procedure, although future work should consider adapting task instructions to minimise the verbal component, as it is possible that the extent of dialogue was difficult for the DD children.

It is also the case that VMA was only measured using the BPVS. This is in keeping with studies within the ASD literature (e.g. Allen and Chambers 2011; Lee and Hobson 2006; Leekam et al. 1998). However, the BPVS only measures single word receptive vocabulary and it is unknown if the groups were matched on skills such as pragmatic skills, grammar and expressive vocabulary. Future research should aim to measure additional aspects of language than simply receptive language comprehension, in order to tease apart whether other skills are facilitating function bias understanding in the other two groups, relative to the DD children.

A further possibility is that the DD children show a fundamental impairment in understanding what objects were made for. Some research supports this proposal. For example, children with intellectual impairment are able to sort objects into categories (Ungerer and Sigman 1987), but they actually perform worse than TD children and children with ASD for superordinate level category matching, particularly for artifactual categories (Tager-Flusberg 1985). This may pervade other areas of language development, including categorisation and play. Thus, clinical and educational programmes should account for this potential problem. It is also conceivable that differences in information processing abilities amongst individuals with DD (Sperber and McCauley 1984), which we did not directly measure, underlie the difficulty the DD group had with understanding the task. There is evidence that individuals with intellectual impairment do not spontaneously abstract relations between pairs of objects (see Paour 1992), and have specific difficulties in working memory (Numminen et al. 2002).

The results of the DD group implicate cognitive delay as the primary source of function bias failure, and based upon the cognitive abilities of our ASD sample, we would also expect impairment across the board in this group. However, our children with ASD were able to pass this task and logistic regression analysis suggests that, in this group, a lower receptive language facilitated performance. Why were children with ASD able to match according to function, while their peers with DD appeared unable to do so? It could be the case that the task was set up to allow low level processes to operate in the ASD sample. Repeatedly emphasising and clearly demonstrating the object’s function may have facilitated function bias understanding.

We can think of several other reasons to explain this finding. First, children with ASD engage in functional play (Baron-Cohen 1987; Jarrold et al. 1993; Libby et al. 1998), which necessitates understanding of an object’s true or intended function (i.e. flying a toy helicopter in the air). Another potential explanation is that our findings reflect a specific strength in ASD during a critical early period of development. Shah and Frith (1983) identified ‘islets of ability’ in ASD in terms of relative strengths in block design tasks. It may be the case that during the earlier stages of language acquisition, children with ASD focus heavily upon the features of objects, and given the rigorous nature and reinforcement of some early intervention programs (Anderson et al. 1987; Lovaas 1987; Vernon et al. 2012), also pay special attention to an adult’s instruction.

Of course, there are limitations to our work. Although we did not find differences between trials that incorporated completely novel objects relative to those that retained some familiarity, future work should utilise a uniform set of stimuli. It would also be advantageous to test more verbally able children with ASD, to generalise our findings across the spectrum and determine whether the function bias is present in individuals whose CA is on a par with their VMA. We advise caution in interpreting our findings because although we found a significant interaction specifically accounted for by the differences between children with ASD and TD children using logistic regression, the interaction revealed by the linear mixed effects model appears to be driven by the stronger association between VMA and function bias scores in TD children. A final limitation is that our DD group included a wide variety of conditions, and future research should aim to explore the function bias in a more homogeneous sample, such as a whole cohort of children with Down Syndrome or a whole cohort of children with intellectual disabilities. This will help tease apart whether subgroups of DD children show the function bias or a function bias deficit is widespread among DD children.

Despite these limitations, our study was the first to investigate the function bias in atypically developing children. Thus, it provides a basis for further work exploring the role of functional information versus shape-based generalisations across development. That children with ASD appear to show the function bias where matched controls with DD do not merits further and deeper investigation.

## Footnotes

1 One child with ASD had a raw score on the BPVS slightly below the basal start point of 2.33. However, as he scored 14 on the BPVS, where a raw score of 15 equates to a VMA of 2.33, this child was conservatively assigned a VMA of 2.25 based upon his raw score.
